# Development of a Drum Tower Severity Scoring (DTSS) system for pyrrolizidine alkaloid-induced hepatic sinusoidal obstruction syndrome

**DOI:** 10.1007/s12072-021-10293-5

**Published:** 2022-01-12

**Authors:** Xuan Wang, Wei Zhang, Ming Zhang, Feng Zhang, Jiangqiang Xiao, Qin Yin, Hao Han, Taishun Li, Ge Lin, Yuzheng Zhuge

**Affiliations:** 1 Department of Gastroenterology, Nanjing Drum Tower Hospital, The Affiliated Hospital of Nanjing University Medical School, 321#, Zhongshan Road, Nanjing, 210008 Jiangsu China; 2 Department of Ultrasound, Nanjing Drum Tower Hospital, The Affiliated Hospital of Nanjing University Medical School, 321#, Zhongshan Road, Nanjing, 210008 Jiangsu China; 3 Medical Statistical Analysis Center, Nanjing Drum Tower Hospital, The Affiliated Hospital of Nanjing University Medical School, 321#, Zhongshan Road, Nanjing, 210008 Jiangsu China; 4 School of Biomedical Sciences, Faculty of Medicine, The Chinese University of Hong Kong, Hong Kong, People’s Republic of China

**Keywords:** Pyrrolizidine alkaloid-induced hepatic sinusoidal obstruction syndrome, Hepatic veno-occlusive disease, Anticoagulation, Prognosis, Disease severity, Acute portal hypertension, Portal vein velocity, Drum Tower Severity Scoring System, Gynura segetum, Drug-induced liver injury

## Abstract

**Background and aims:**

There has been no reliable severity system based on the prognosis to guide therapeutic strategies for patients with pyrrolizidine alkaloid (PA)-induced hepatic sinusoidal obstruction syndrome (HSOS). We aimed to create a novel Drum Tower Severity Scoring (DTSS) system for these patients to guide therapy.

**Methods:**

172 Patients with PA-HSOS who received supportive care and anticoagulation therapy in Nanjing Drum Tower Hospital from January 2008 to December 2020 were enrolled and analyzed retrospectively. These patients were randomized into a training or validation set in a 3:1 ratio. Next, we established and validated the newly developed DTSS system.

**Results:**

Analysis identified a predictive formula: logit (*P*) = 0.004 × aspartate aminotransferase (AST, U/L) + 0.019 × total bilirubin (TB, μmol/L) − 0.571 × fibrinogen (FIB, g/L) − 0.093 × peak portal vein velocity (PVV, cm/s) + 1.122. Next, we quantified the above variables to establish the DTSS system. For the training set, the area under the ROC curve (AUC) (*n* = 127) was 0.787 [95% confidence interval (CI) 0.706–0.868; *p* < 0.001]. With a lower cut-off value of 6.5, the sensitivity and negative predictive value for predicting no response to supportive care and anticoagulation therapy were 94.7% and 88.0%, respectively. When applying a high cut-off value of 10.5, the specificity was 92.9% and the positive predictive value was 78.3%. For the validation set, the system performed stable with an AUC of 0.808.

**Conclusions:**

The DTSS system can predict the outcome of supportive care and anticoagulation in PA-HSOS patients with satisfactory accuracy by evaluating severity, and may have potential significance for guiding therapy.

**Supplementary Information:**

The online version contains supplementary material available at 10.1007/s12072-021-10293-5.

## Introduction

Herbal medicine and various dietary supplements are both common causes of drug-induced liver injury (DILI) worldwide. Recently, pyrrolizidine alkaloid (PA)-induced hepatic sinusoidal obstruction syndrome (PA-HSOS) has aroused widespread concern because of its increasing incidence [1]. In contrast to other forms of DILI, PA-HSOS is characterized by acute portal hypertension accompanied by abnormal liver function; this results from the formation of blood flow stasis in the sinus caused by edema, necrosis, and exfoliation of endothelial cells in the hepatic sinusoid, hepatic venule, and interlobular vein [2]. Besides abnormal liver function, these patients usually experience abdominal distention, ascites, jaundice, and abdominal pain [3]. For a considerable period of time, the only supportive therapy available for these patients was the use of diuretics to reduce abdominal distention; the mortality rate ranges over 12.5–70%. However, recent research has suggested that anticoagulation therapy is effective in approximately 50% of PA-HSOS patients [4].

In recent years, an increasing evidence has led to the consensus for the efficacy of anticoagulation therapy in PA-HSOS [5–7]. For patients with no response to anticoagulation, the overall response rate to subsequent trans-jugular intrahepatic portosystemic shunt (TIPS) therapy was 91% [4]. Therefore, anticoagulation-TIPS ladder treatment is the recommended therapeutic strategy for patients with PA-HSOS at present [5].

Research has shown that PA-HSOS patients with mild or moderate severity grades, based on the severity criteria for sinusoidal obstruction syndrome published by the European Blood and Bone Marrow Transplantation (EBMT) Collaboration Group in 2016 [8], are more likely to benefit from anticoagulation therapy. However, 30.17% of mild patients and 33.82% of moderate patients were reported to be unresponsive [9]. The severity criteria put forward by the EBMT Collaboration Group is associated with certain shortcomings. For example, HSOS induced by either chemotherapy drug pretreatment or PA exposure is essentially a form of vascular liver disease that is characterized by acute portal hypertension. Accordingly, the absence of portal vein hemodynamic parameters in the severity classification creates a significant defect that may lead to reduced sensitivity when evaluating severe patients. Second, PA-HSOS is a sporadic condition; it is difficult to obtain an accurate pre-onset weight. Third, PA-HSOS can be easily misdiagnosed due to the lack of specific clinical characteristics and the sporadic nature of this disease, thus leading to an artificially prolonged period of the time from the onset of symptoms to diagnosis. Fourth, some patients are treated with large doses of diuretics or by the repeated drainage of ascites before diagnosis; hence, elevated serum creatinine (Scr) may be an iatrogenic effect rather than the result of kidney damage arising from the disease. In consideration of these defects, in this study, we developed and validated a more stable severity grading system, the Drum Tower Severity Scoring (DTSS) system, to evaluate the severity of PA-HSOS patients, so as to better guide clinical treatment and improve the prognosis of disease.

## Methods

### Patient selection

Suspected PA-HSOS patients admitted to our center between January 2008 and December 2020 were retrospectively evaluated for eligibility, it should be noted that the pyrrolizidine alkaloid-containing plant administered to the patients enrolled in this study was Tu-San-Qi (Gynura japonica), a plant most commonly known to cause the disease in China [1]. The follow-up period was from January 2008 to March 2021. The inclusion criteria were as follows: (1) diagnosed as PA-HSOS based on the Nanjing criteria for PA-HSOS [5], (2) receiving anticoagulation-based medication, and (3) aged between 18 and 85 years. The exclusion criteria were as follows: (1) the presence of more than two types of liver damage factors, such as alcohol consumption, hepatophilic virus infection, and autoimmune liver diseases, or cirrhosis-induced portal hypertension existed before intaking herb containing PA [10], (2) multiple organ dysfunction (MODS) identified prior to anticoagulation therapy, (3) malignant tumors, (4) incomplete datasets, and (5) unknown anticoagulation outcome.

### Ethical approval

This study was registered (ChiCTR2100050417), approved by the ethic committees of Nanjing Drum Tower Hospital and conformed to the principles of the World Medical Association Declaration of Helsinki, as revised in 2013.

### Informed consent

This study was a retrospective study, so the authors requested and received permission to waive informed consent.

### Sample size calculation

This study was a diagnostic test with a power of 0.80 and an alpha of 0.05. Based on the fact that the overall complete remission rate was 60.9% in PA-HSOS patients receiving anticoagulation therapy [9], the prevalence (no response to anticoagulation rate) in our study was 0.391. With a Se1 (alternative sensitivity) of 0.95, a Sp1 (alternative specificity) of 0.90, both Se0 (null sensitivity) and Sp0 (null specificity) equivalent to 0.75, the minimum sample size required was 89 cases according to Power Analysis and Sample Size (PASS) (NCSS statistical software, Kaysville, Utah, USA) version 15.0. The sample size used to build the DTSS system in the training set included 127 cases.

### Statistical analysis

Statistical analysis was performed by IBM SPSS Statistics version 23.0 (IBM, Armonk, New York, USA), R studio version 4.0.4, and GraphPad Prism 8 (GraphPad Software, La Jolla, California, USA). Data were tested for normality with the Kolmogorov–Smirnov test. Normal continuous variables (*p* > 0.1) are described as means ± standard deviation (SD) and were compared by an independent samples *t* test. Non-normal continuous variables are described as medians and interquartile ranges (IQR) and were compared with the Mann–Whitney *U* test. Categorical variables are presented as numbers (*N*) with percentages (%) and were analyzed with the Chi-squared or Fisher's exact test. In total, 172 patients were randomly divided into the training set (*n* = 129) and validation set (*n* = 43) in a 3:1 ratio. In the training set, we evaluated several related clinical indexes by logistic regression analysis, identified independent predictors by multiple logistic regression analysis (Enter method), and further obtained their own predictive efficacy by receiver operating characteristic (ROC) analysis. We established several models by multiple logistic regression analysis (forward and backward stepwise LR methods, or choosing them by considering with their clinical implications), and evaluated the efficacy of each model by performing ROC analysis in different subgroups. Finally, we constructed a new scoring system according to the best model to evaluate anticoagulation efficacy and defined appropriate cut-off values according to a sensitivity of 95% and a specificity of 90%. *p* < 0.05 indicated significant statistical differences.

### Evolutionary approach of the DTSS system

Among the candidate models, we gave priority to overall effectiveness [AUC and 95% confidence interval (CI)] and selected the best model with a small number of indicators and good objectivity. The metrics of the best model were then quantified to simplify clinical application. The quantification was based on model coefficients, normal upper limit of indicators, results of ROC analysis for single indicator’s prediction of anticoagulation, definition of invalid anticoagulation [total bilirubin (TB) levels > 5 mg/dL, peak portal vein velocity (PVV) < 10 cm/s], and European [fibrinogen (FIB) < 0.8–1.0 g/L] and American (FIB < 1.5–2.0 g/L) Society of Anesthesiologists indications for perioperative fibrinogen infusion [11, 12].

### Definition

#### Peak PVV detection

Patients were required to fast for at least 8 h prior to examination and the operators were all ultrasound specialists with at least 3 years of related experience. The Siemens Acuson S2000 or S3000, 4C1 convex array probe was used to measure the internal diameter and the highest blood velocity at 1.0–2.0 cm from the portal vein trunk bifurcation. The volume and angle of Doppler sampling needed to be adjusted according to the anatomy and inner diameter of the blood vessel, so that the angle between the sound beam and the blood flow beam was ≤ 60°. For Color Doppler Flow Imaging (CDFI) examination, we turned down the flow velocity range, turned up the color gain, and lowered the wall filter. While measuring flow velocity by spectral Doppler, we turned down the velocity scale.

### Anticoagulation therapy

For PA-HSOS patients without active bleeding or high-risk factors of bleeding, anticoagulation therapy was chosen on the basis of diuretic and liver protective treatment, including low-molecular-weight heparin alone [for patients with a contraindication to warfarin or a baseline international normalized ratio (INR) > 1.5, 4000 IU/bid, ih], low-molecular-weight heparin together with warfarin [indication: INR < 1.5 and platelets (PLT) > 50 × 10^9^/L], or suitable dose of warfarin (indication: patients whose PLT < 50 × 10^9^/L or who showed high sensitivity to heparin, starting with 1.5 mg). The INR was maintained at 2–3 [5].

### Efficacy determination

No response was defined as (1) either TB ≥ 5 mg/dL, or peak PVV < 10 cm/s, or (2) any two of the following four were met during 2 weeks anticoagulation: no improvement of TB (< 5 mg/dL), increment less than 10% of peak PVV of baseline, no significant relief of ascites, concurrent renal and coagulation deterioration. These patients were considered as non-responders to anticoagulation therapy and were recommended to receive TIPS treatment [13]. Patients who experienced relief from ascites, along with no deterioration in TB and peak PVV from baseline, were regarded as responders to anticoagulation and asked to maintain anticoagulation therapy until clinical curation.

### Disease course

Acute onset was defined as the occurrence of clinical symptoms < 35 days after starting PAs. In contrast, the chronic onset of disease referred to the onset of symptoms ≥ 35 days starting PAs.

### Ascites grading

Grade 1 ascites were detected in multiple spaces at a depth of less than 3 cm under ultrasound. Grade 2 ascites were detected by ultrasound at a depth of 3–10 cm. Grade 3 ascites were detected by ultrasound and occupied the entire abdominal cavity at a depth > 10 cm [14].

## Results

### Patient characteristics

**Fig. 1:**
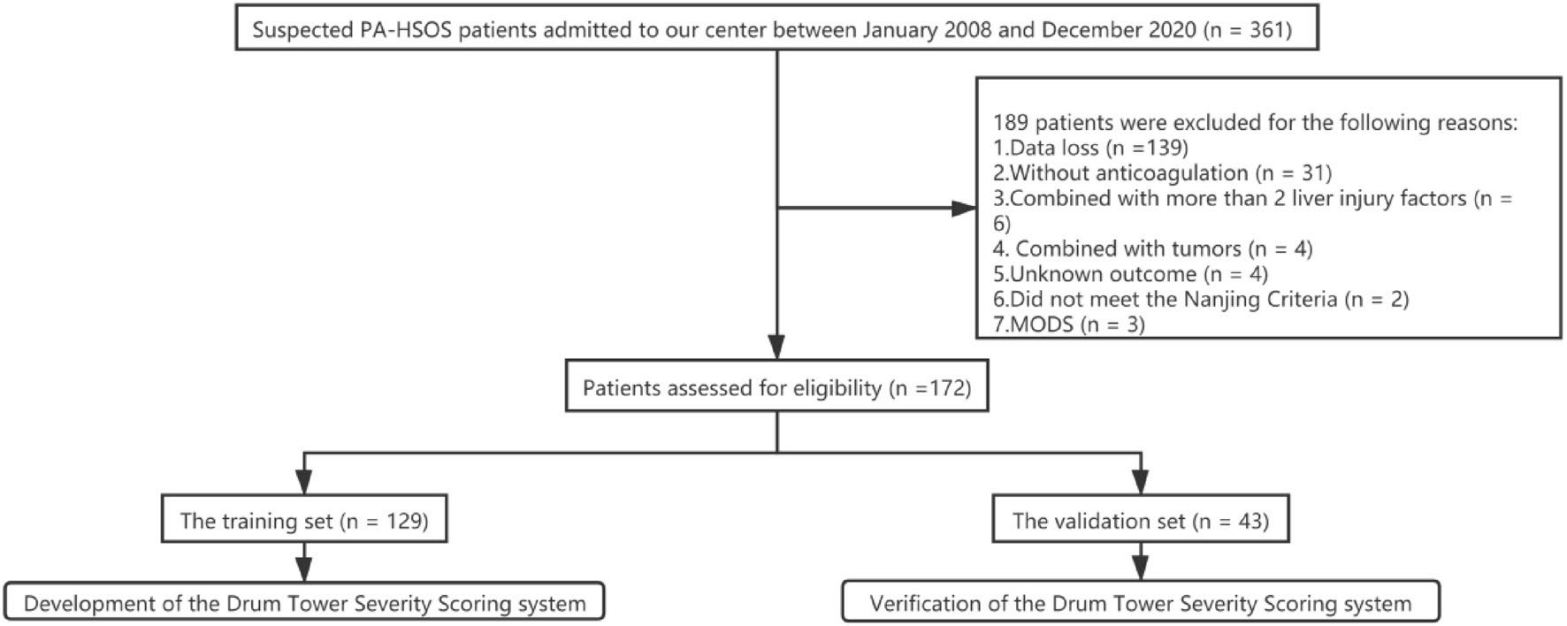
Flow chart depicting the flow of work used in this study

In total, we recruited 172 PA-HSOS patients aged between 36 and 83 years, including 100 males and 72 females. According to different anticoagulation outcomes, patients were divided into a response group (*n* = 85) and a non-response group (*n* = 87). 49 cases had liver biopsies in this study, including 20 cases in the response group and 29 cases in the non-response group. All cases met the typical manifestations of HSOS (hepatic sinusoidal stasis and dilatation), and there were no difference in subgroups. In summary, the concordance rate of the 49 liver biopsies with the Nanjing criteria was 100%, which was in line with our previous research [15]. In the no response group, 80 patients received TIPS and the remaining seven died (without TIPS). One patient died from gastrointestinal bleeding after 2 weeks of anticoagulation, three patients died due to multiple organ dysfunction, two patients died after 3 months of anticoagulation due to liver failure, and the last patient died from unknown causes. No patients received liver transplantation. Figure 1 shows a flow chart depicting the process used to screen patients.

### Predictor screening

When analyzing baseline indicators in the response group and the non-response group, we identified significant statistical differences for 11 indicators, as detailed in Table 1. We considered the following 12 variables as potentially associated with anticoagulant outcome: course of disease (*p* = 0.034), time from onset to anticoagulation (*p* = 0.548), alanine aminotransferase (ALT, *p* = 0.017), aspartate aminotransferase (AST, *p* = 0.001), TB (*p* < 0.001), albumin (ALB, *p* = 0.261), Scr (*p* = 0.021), prothrombin time (PT, *p* < 0.001), FIB (*p* = 0.02), D2 polymers (D2, *p* < 0.001), peak PVV (*p* < 0.001), and portal vein thrombosis (PVT, *p* = 0.578). In the training set (*n* = 129), we confirmed that five variables had predictive value (*p* < 0.05): AST, TB, PT, FIB and peak PVV; TB and peak PVV were both independent predictors, as shown in Table 2. Interestingly, these indices exhibited poor abilities to predict anticoagulation outcome when considered alone (AUC < 0.700, *p* < 0.05), as shown in the Supportive Materials.

**Table 1:** Baseline characteristics

Variable	Total (*n* = 172)	Valid (*n* = 85)	Invalid (*n* = 87)	*p* value
Gender (male: *N*, %)	100 (58.1%)	43 (50.6%)	57(65.5%)	0.047
Age (year: median, IQR)	65 (60, 71)	64 (60, 71)	66 (62, 70)	0.623
Course (acute: *N*, %)	99 (57.6%)	56 (66.7%)	43 (50.6%)	0.034
Diabetes (*N*, %)	23 (13.4%)	9 (10.6%)	14 (16.1%)	0.289
Hypertension (*N*, %)	77 (44.8%)	39 (45.9%)	38 (43.7%)	0.771
Coronary heart disease (*N*, %)	7 (4.1%)	6 (7.1%)	1 (1.1%)	0.063
History of liver disease (*N*, %)	8 (4.7%)	4 (4.7%)	4 (4.6%)	1.000
Alcohol (*N*, %)	51 (29.7%)	20 (23.5%)	31 (35.6%)	0.082
*Mode of PA intake (N, %)*				0.275
1. Water	86 (50.0%)	48 (56.5%)	38 (43.7%)	
2. Wine	45 (26.2%)	17 (20.0%)	28 (32.2%)	
3. Powder	19 (11.0%)	9 (10.6%)	10 (11.5%)	
4. Unknown	22 (12.8%)	11 (12.9%)	11 (12.6%)	
Time from intake to onset (day: median, IQR)	31 (14, 61)	28.5 (13.25, 46.75)	31 (15.5, 92)	0.029
Time from onset to diagnosis (day: median, IQR)	24 (14.25, 32)	27 (16, 39)	21 (14, 31)	0.098
Time from onset to anticoagulation (day: median, IQR)	30 (16.25, 44)	30 (17.5, 45.5)	30 (16,44)	0.548
Time from anticoagulation to TIPS (day: median, IQR)	12 (5.25, 20)	/	12 (5.25, 20)	
PLT (10^9^/L: median, IQR)	106 (80, 141.75)	110 (82.5, 146)	102 (71, 139)	0.165
ALT (U/L: median, IQR)	55.7 (29.425, 139.4)	45.6 (29, 89.5)	71.7 (35.6, 190)	0.017
AST (U/L: median, IQR)	76.7 (47.85, 119.15)	64.1 (45.15, 92.3)	89.4 (54.6, 183.2)	0.001
TB (µmol/L: median, IQR)^1^	36.45 (25.325, 54.45)	32.5 (21.7, 45.85)	42.9 (30.8, 67.6)	0.000
ALB (g/L: mean ± SD)	32.918 ± 3.6103	33.232 ± 3.6993	32.611 ± 3.5155	0.261
Scr (µmol/L: median, IQR)	70.5 (60, 87)	68 (59, 81)	74 (62, 101)	0.021
CRP (mg/L: median, IQR)	13.65 (7.8, 26.925)	12.6 (7.2, 19.6)	15.4 (8.2, 29.2)	0.206
PT (s: median, IQR)	14.9 (13.7, 16.675)	14.4 (13.25, 15.25)	15.6 (14.3, 17.2)	0.000
D2 (g/L: median, IQR)	2.2 (1.8, 2.7)	1.45 (0.923, 2.848)	2.19 (1.335, 3.105)	0.000
FIB (g/L: median, IQR)^2^	1.92 (1.015, 2.93)	2.4 (2, 2.9)	1.9 (1.5, 2.5)	0.020
Peak PVV (cm/s: mean ± SD)^3^	14.708 ± 6.1358	16.409 ± 6.2124	13.047 ± 5.6134	0.000
HVPG (mmHg: mean ± SD)	20.4129 ± 5.34179	19.2323 ± 3.96053	21.3748 ± 6.15385	0.165
PVT (*N*, %)	12 (7.0%)	5 (6.2%)	7 (8.4%)	0.578
*Ascites grade (N, %)*				0.533
Non	1 (0.6%)	0 (0%)	1 (1.1%)	
Grade 1 (mild)	4 (2.3%)	2 (2.4%)	2 (2.3%)	
Grade 2 (moderate)	123 (71.5%)	58 (68.2%)	65 (74.7%)	
Grade 3 (severe)	44 (25.6%)	25 (29.4%)	19 (21.8%)	

^a^TB: the normal range of TB in our center is < 20.5 µmol/L

^b^FIB: to note, two cases in the valid group had missing data (compared to three cases in the invalid group)

^c^Peak PVV: the normal range of peak PVV is ≥ 20 cm/s

**Table 2:** Logistic regression (the training set)

Variable	Univariate			Multivariate (enter method)		
	*B*	OR 95%CI	*p*	*B*	OR 95%CI	*p*
Acute onset	− 0.383	0.682 [0.334, 1.393]	0.294			
Time from onset to anticoagulation	− 0.009	0.991 [0.997, 1.005]	0.209			
ALT	0.003	1.003 [1.000, 1.005]	0.056			
AST	0.005	1.005 [1.001, 1.009]	0.012			
TB	0.029	1.030 [1.012, 1.048]	0.001	0.031	1.032 [1.004, 1.061]	0.026
ALB	− 0.047	0.955 [0.869, 1.049]	0.333			
Scr	0.008	1.008 [0.997, 1.021]	0.166			
PT	0.318	1.374 [1.140, 1.656]	0.001			
D2	0.106	1.112 [0.933, 1.324]	0.235			
FIB	− 0.899	0.407 [0.224, 0.741]	0.003			
Peak PVV	− 0.127	0.881 [0.819, 0.947]	0.001	− 0.129	0.879 [0.793, 0.975]	0.015
PVT	0.215	1.240 [0.340, 4.524]	0.745			

### Development of the DTSS system for PA-HSOS

**Fig. 2:**
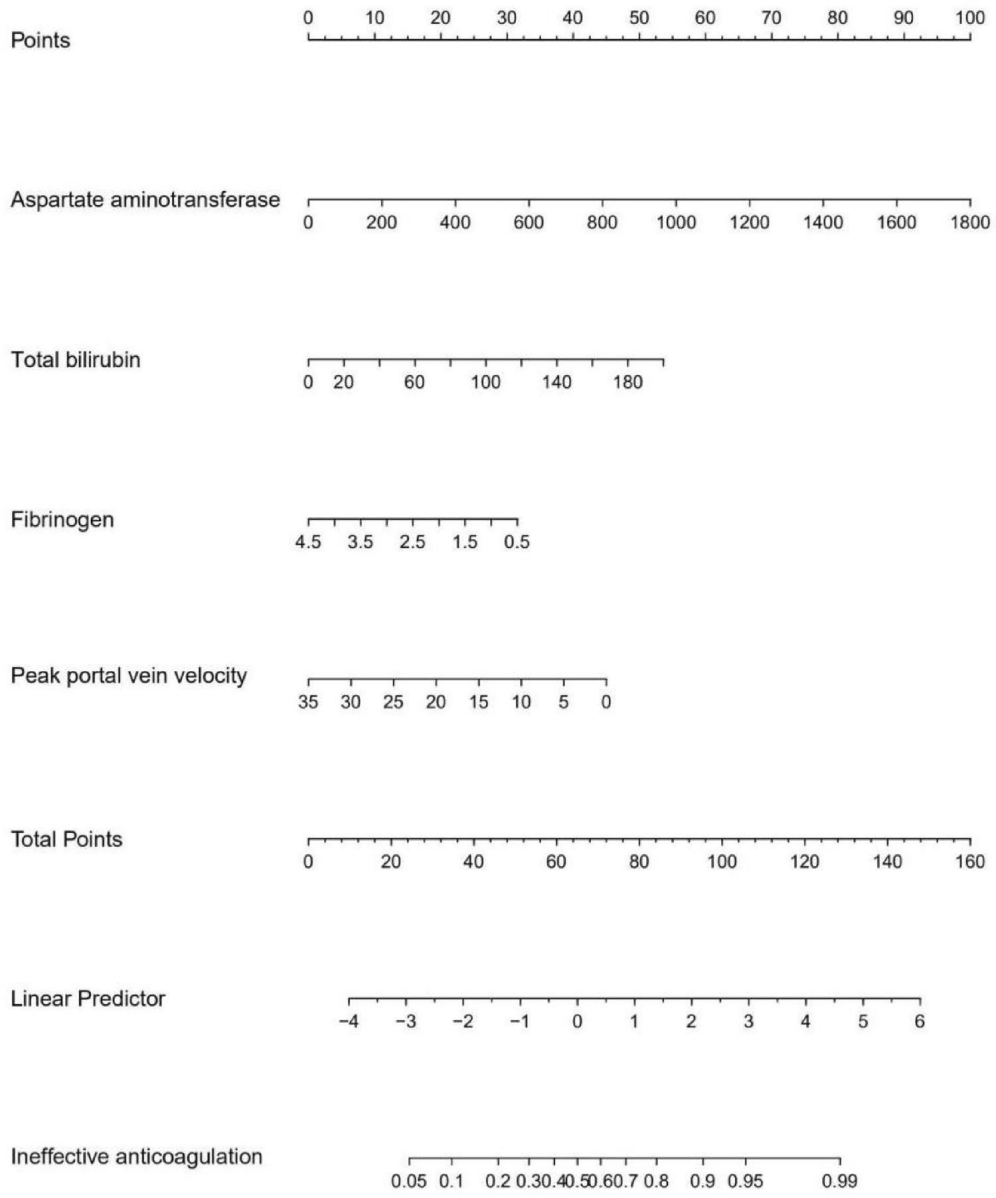
A nomogram for model 3

**Fig. 3:**
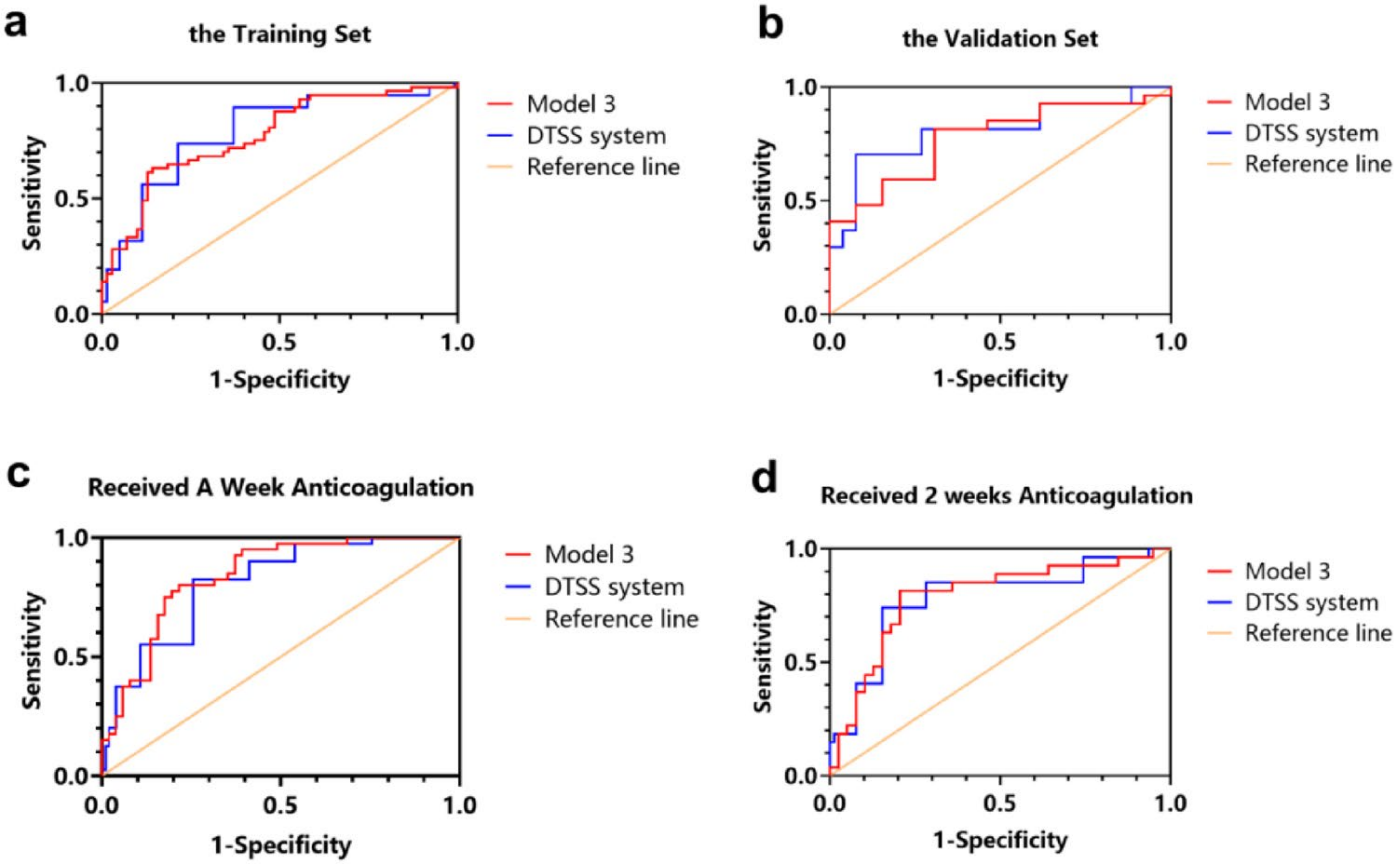
ROC curve for the DTSS system and model 3 in different subgroups. **a** In the training set (*n* = 127), the AUC of model 3 was 0.773, 95% CI [0.691, 0.855], the AUC of the system = 0.787, 95% CI [0.706, 0.868]. **b** In the validation set (*n* = 40), the AUC of model 3 was 0.775, 95% CI [0.628, 0.922], the AUC of the system = 0.808, 95% CI [0.670, 0.946]. **c** Inpatients receiving anticoagulation over one week (*n* = 91), the AUC of model 3 was 0.840, 95% CI [0.760, 0.921], the AUC of the system = 0.812, 95% CI [0.725, 0.898]. **d** Inpatients receiving 2 weeks of anticoagulation (*n* = 66), the AUC of model 3 was 0.789, 95% CI [0.672, 0.906], the AUC of the system was 0.783, 95% CI [0.663, 0.902]

In the training set, we evaluated the efficacy of each model and found that model 3 was both efficient and simple. The formula for model 3 (*n* = 127) was as follows: logit (*P*) = 0.004 × AST (U/L) + 0.019 × TB (µmol/L) − 0.571 × FIB (g/L) − 0.093 × peak PVV (cm/s) + 1.122, AUC = 0.773, 95% CI 0.691–0.855, *p* < 0.001. The nomogram for model 3 is shown in Fig. 2. On the basis of model 3, and to facilitate clinical application, we designed model 5 (the DTSS system) which had an AUC = 0.787, 95% CI 0.706–0.868, *p* < 0.001. In this system, we found that when the low cut-off value was 6.5, the sensitivity to exclude non-responsive patients was 94.7%, and the negative predictive value (NPV) was 88.0%. When the high cut-off value was 10.5, the specificity of predicting an invalid anticoagulation outcome was 92.9% and the positive predictive value (PPV) was 78.3%. In conclusion, patients with a score of 4–6 were defined as mild, patients with a score of 7–10 were defined as moderate, and patients with a score of 11–16 were defined as severe. All data are shown in Tables 3, 4 and 5 and Fig. 3.

**Table 3:** Performance of different models in subgroups

Model	The training set (before anticoagulation)			The validation set (before anticoagulation)			Revisited patients (1 week anticoagulation)			Revisited patients (2 weeks anticoagulation)		
	AUC (95% CI)	SE	*p*	AUC (95% CI)	SE	*p*	AUC (95% CI)	SE	*p*	AUC (95% CI)	SE	*p*
① PT + FIB + AST + TB + peak PVV + Scr	0.778 [0.696, 0.859]	0.042	0.000	0.775 [0.631, 0.919]	0.074	0.005						
② AST + TB + peakPVV	0.768 [0.686, 0.849]	0.042	0.000	0.718 [0.561, 0.875]	0.080	0.022						
③ FIB + AST + TB + peak PVV	0.773 [0.691, 0.855]	0.042	0.000	0.775 [0.628, 0.922]	0.075	0.005	0.840 [0.760, 0.921]	0.041	0.000	0.789 [0.672, 0.906]	0.060	0.000
④ PT + FIB + AST + TB + peak PVV	0.775 [0.693, 0.856]	0.042	0.000	0.775 [0.629, 0.920]	0.074	0.005						
⑤ DTSS system	0.787 [0.706, 0.868]	0.041	0.000	0.808 [0.670, 0.946]	0.070	0.002	0.812 [0.725, 0.898]	0.044	0.000	0.783 [0.663, 0.902]	0.061	0.000

*SE* standard error

**Table 4:** Drum Tower Severity Scoring (DTSS) system

Variable	1 point	2 point	3 point	4 point
AST(U/L)	< 69.75	≥ 69.75, < 200	≥ 200, < 320	≥ 320
TB (µmol/L)	< 20.5	≥ 20.5, < 38	≥ 38, < 85.5	≥ 85.5
FIB (g/L)	> 2.35	> 1.5, ≤ 2.35	> 1, ≤ 1.5	≤ 1
Peak PVV (cm/s)	≥ 20	> 15.85, < 20	≥ 10, ≤ 15.85	< 10

**Table 5:** Diagnostic performance between DTSS system and revised EBMT criteria

	Cut-off values	Ture positive (a)	False positive (b)	Ture negative (d)	False negative (c)	Sensitivity (%)	Specificity (%)	PPV (%)	NPV (%)
Training set (DTSS, *n* = 127)	6.5	54	48	22	3	94.7	31.4	52.9	88
	10.5	18	5	65	39	31.6	92.9	78.3	62.5
Validation set (DTSS, *n* = 40)	6.5	25	10	3	2	92.6	23.1	71.4	60
	10.5	8	0	13	19	29.6	100	100	40.6
One week Anticoagulation (DTSS, *n* = 91)	6.5	36	24	27	4	90	52.9	60	87.1
	10.5	8	1	50	32	20	98.0	88.9	61.0
Two weeks anticoagulation (DTSS, *n* = 66)	6.5	23	15	24	4	85.2	61.5	60.5	85.7
	10.5	4	0	39	23	14.8	100	100	62.9
Before anticoagulation (EBMT^a^, *n* = 172)	1.5	42	37	48	45	48.3	56.5	53.2	51.6
	2.5	20	4	81	67	23.0	95.3	83.3	54.7

^a^We redefined the modified EBMT classification: 1 for mild, 2 for moderate, 3 for severe, and 4 for very severe

### Validation of the DTSS system for PA-HSOS

In the validation set, the AUC for model 3 (*n* = 40) was 0.775, with a 95% CI of 0.628–0.922, *p* = 0.005, and the AUC for the DTSS system was 0.808, with a 95% CI of 0.670–0.946, *p* = 0.002. For 91 patients who underwent anticoagulation therapy for more than a week, these two models still had considerable evaluation value (model 3: AUC = 0.840, 95% CI 0.760–0.921, *p* < 0.001; DTSS system: AUC = 0.812, 95% CI 0.725–0.898, *p* < 0.001). For 66 patients with 2 weeks of anticoagulation treatment, similar results were obtained (model 3: AUC = 0.789, 95% CI 0.672–0.906, *p* < 0.001; DTSS system: AUC = 0.783, 95% CI 0.663–0.902, *p* < 0.001). In addition, we classified 172 patients as mild, moderate, and severe or greater, by the revised EBMT criteria, and tested its efficiency. We found that compared with the DTSS system, the existing criteria had worse sensitivity (48.3% vs 94.7%) and NPV (51.6% vs 88%), while the specificity (95.3% vs 92.9%) and PPV (83.3% vs 78.3%) were similar. All data are shown in Tables 3, 4 and 5 and Fig. 3.

## Discussion

In the West, HSOS is often observed following the high-dose chemotherapy used in hematopoietic stem cell transplantation (HSCT). Toxic metabolites arising from chemotherapy used in HSCT can lead to the injury of sinusoidal endothelial cells and hepatocytes, and the obstruction of the hepatic sinusoids and terminal hepatic veins, thus promoting the development of hepatic dysfunction and acute portal hypertension [16, 17]. Previous studies have shown that a history of prior liver disease and hematologic disorders are high risk factors for HSOS progression [8]. However, in China and Southeast Asia, the main cause of HSOS is patients consuming plants containing pyrrolizidine alkaloids (PAs) [18]. In contrast to inpatients with HSCT-HSOS, patients with PA-HSOS do not generally have any underlying diseases; in most cases, the disease is sporadic. Therefore, it is very difficult to obtain accurate and complete parameters from such patients, such as changes in body weight and the serum levels of total bilirubin; these are very important parameters in the revised EBMT criteria [19]. Another significant feature found in PA-HSOS patients was that 70–80% of patients in the early stages of disease showed only mild elevations in bilirubin of 1–3 mg/dL; the change in serum bilirubin was also very slow, although the hepatic venous pressure gradient (HVPG) was extremely high during the early stages of disease [4]. Notably, despite the administration of anticoagulation therapy in the early stages of disease, approximately 40–50% of patients gradually deteriorated and eventually converted to severe PA-HSOS. The regularity of this change in disease status may lead to insufficient sensitivity with regards to the revised EBMT criteria to judge the severity of the disease in its early stages, thus leading to the inaccurate prediction of disease prognosis. Considering the clear defects in the revised EBMT criteria, we believe that it is necessary to improve the HSOS severity grading criteria so as to provide better sensitivity in predicting patient outcomes in the early stages of disease. In this study, we established a new HSOS severity grading system, the DTSS system for PA-HSOS, based on our PA-HSOS patient cohort, and verified this new system internally.

It is always well known that the key difference between HSOS and other common forms of DILI is that the endothelial cells of the blood sinus are the primary site of injury in HSOS, and acute portal hypertension is its prominent clinical feature. Numerous studies have shown that TIPS can reverse the deterioration of disease in PA-HSOS patients who are unresponsive to anticoagulation therapy and ultimately improve survival [20, 21]. These phenomena suggest that portal hypertension is a key factor that affects the prognosis of patients with PA-HSOS and that portal hemodynamic parameters may be associated with the severity and prognosis of the disease. In addition, considering that drastic changes in the portal vein hemodynamics of patients with PA-HSOS are early events of the disease, it follows that the parameters related to portal vein hemodynamics should be more sensitive to reflect the severity of PA-HSOS than either serum bilirubin and creatinine. Therefore, it is reasonable to incorporate portal vein hemodynamic indicators into the PA-HSOS severity grading system. It is not beyond our expectations that patients who did not respond to anticoagulant had a significant lower peak PVV as compared to patients who did respond to anticoagulant, and that peak PVV was the other independent factor affecting the prognosis of anticoagulant therapy besides serum bilirubin in our current multivariate analysis. Another key advantage to this is that peak PVV, as a critical parameter, can be obtained readily in a non-invasive manner.

Fibrinogen is associated with blood coagulation status and liver function, but is less affected by anticoagulation therapy than PT. Even though serum creatinine is an important parameter in the revised EBMT criteria, it did not improve the efficacy of the model developed in this study, because the effectiveness of model 1 and model 4 was similar. We speculated that although the deterioration of renal function in PA-HSOS patients was associated with the progression of acute portal hypertension and massive ascites, the excessive diuresis and drainage of ascites may also lead to damage to renal function, thus exaggerating the severity of the condition. In line with our speculation, our results showed that compared with our new DTSS system for PA-HSOS, the revised EBMT criteria performed poorly in terms of both sensitivity (48.3%) and NPV (51.6%).

To screen patients who were non-responsive to anticoagulation and guide subsequent treatment, we used a sensitivity of 95% and a specificity of 90% as cut-off thresholds. In the training set, we observed that patients defined as being mild by the DTSS system (scores of 4–6) had an 88% probability of effective anticoagulation. Patients defined as being severe by the system (11–16 points) had a 78.3% probability of non-response to anticoagulation. The direct use of TIPS treatment for these patients may be a more reasonable treatment regimen. Although patients defined as moderate (7–10 points) had a 45.6% probability of ineffective anticoagulation, in the context of this situation, anticoagulation and support therapy should be performed under close observation, and timely TIPS intervention should be considered once the medication fails.

The major limitation of the present study was the absence of a prospective cohort for external validation. Furthermore, we did not include blood pyrrole serum protein adducts (PPA), the PA-derived specific biomarker, to the system from an etiological point-of-view. In a previous study, Gao et al. reported that PPAs levels in severe PA-HSOS patients (those who died or received liver transplantation) were significantly higher than those in mild patients (recovery or chronicity in the end) (*p* = 0.004) [22]. Notably, recently their research group also developed new biomarkers, such as blood pyrrole–hemoglobin adducts (PHA) and urinary pyrrole–amino acid adducts (PAAA) for definitive and non-invasive diagnosis of PA-HSOS [23]. To better diagnose and estimate the severity of PA-HSOS and design more successful therapy, further correlation tests between these PA-derived biomarkers and clinical manifestations of PA-HSOS are warranted.

In conclusion, the Drum Tower Severity Scoring (DTSS) system can predict the effect of anticoagulation in PA-HSOS patients, with satisfactory levels of accuracy by evaluating the severity of disease. We recommend outpatient anticoagulation for patients with scores of 4–6 and follow-up every other week. Patients with scores of 7–10 should be hospitalized and monitored weekly. Direct TIPS is recommended for patients with scores of 11–16.

## Supplementary Information

Below is the link to the electronic supplementary material.

Supplementary file1 (ZIP 60 kb)

Supplementary file2 (RAR 66 kb)

Supporting materials: including missing data and its disposal, a table of ROC analysis between related predictors, a table of RUCAM score to evaluate the drug-induced liver injury (DOCX 16 kb)

## Abbreviations

PLT: Platelets
ALT: Glutathione aminotransferase
AST: Aspartate aminotransferase
TB: Total bilirubin
ALB: Albumin
Scr: Serum creatinine
CRP: C reaction protein
PT: Prothrombin time
D2: D2 aggregates
FIB: Fibrinogen
PVV: Portal vein velocity
HVPG: Hepatic venous pressure gradient
PVT: Portal vein thrombosis
