# Supplementary material for: Development of a Drum Tower Severity Scoring (DTSS) system for pyrrolizidine alkaloid-induced hepatic sinusoidal obstruction syndrome
Source: Hepatol Int. 2022 Jan 12;16(3):669–79. doi: 10.1007/s12072-021-10293-5 (PMC9174127; doi:10.1007/s12072-021-10293-5)
Supplement: Supplementary file 1 — Supplementary file1 (ZIP 60 kb) [file 12072_2021_10293_MOESM1_ESM.zip › Table 1.docx]

Table 1 Baseline characteristics

| **Variable** | **Total (n=172)** | **Valid (n=85)** | **Invalid (n=87)** | **P value** |
| --- | --- | --- | --- | --- |
| **Gender (male: N, %)** | 100 (58.1%) | 43 (50.6%) | 57(65.5%) | 0.047 |
| **Age (year: median, IQR)** | 65 (60, 71) | 64 (60, 71) | 66 (62, 70) | 0.623 |
| **Course (acute：N, %)** | 99 (57.6%) | 56 (66.7%) | 43 (50.6%) | 0.034 |
| **Diabetes (N, %)** | 23 (13.4%) | 9 (10.6%) | 14 (16.1%) | 0.289 |
| **Hypertension (N, %)** | 77 (44.8%) | 39 (45.9%) | 38 (43.7%) | 0.771 |
| **coronary heart disease (N，%)** | 7 (4.1%) | 6 (7.1%) | 1 (1.1%) | 0.063 |
| **History of liver disease (N，%)** | 8 (4.7%) | 4 (4.7%) | 4 (4.6%) | 1.000 |
| **Alcohol (N, %)** | 51 (29.7%) | 20 (23.5%) | 31 (35.6%) | 0.082 |
| **Mode of PA intake (N, %)** |  |  |  | 0.275 |
| **1.Water** | 86 (50.0%) | 48 (56.5%) | 38 (43.7%) |  |
| **2.Wine** | 45 (26.2%) | 17 (20.0%) | 28 (32.2%) |  |
| **3.Powder** | 19 (11.0%) | 9 (10.6%) | 10 (11.5%) |  |
| **4.Unknown** | 22 (12.8%) | 11 (12.9%) | 11 (12.6%) |  |
| **Time from intake to onset (day: median, IQR)** | 31 (14, 61) | 28.5 (13.25, 46.75) | 31 (15.5, 92) | 0.029 |
| **Time from onset to diagnosis (day: median, IQR)** | 24 (14.25, 32) | 27 (16, 39)) | 21 (14, 31) | 0.098 |
| **Time from onset to anticoagulation (day: median, IQR)** | 30 (16.25, 44) | 30 (17.5, 45.5) | 30 (16,44) | 0.548 |
| **Time from anticoagulation to TIPS (day: median, IQR)** | 12 (5.25, 20) | / | 12 (5.25, 20) |  |
| **PLT (10^9^/L: median, IQR)** | 106 (80, 141.75) | 110 (82.5, 146) | 102 (71, 139) | 0.165 |
| **ALT (U/L: median, IQR)** | 55.7 (29.425, 139.4) | 45.6 (29, 89.5) | 71.7 (35.6, 190) | 0.017 |
| **AST (U/L: median, IQR)** | 76.7 (47.85, 119.15) | 64.1 (45.15, 92.3) | 89.4 (54.6, 183.2) | 0.001 |
| **TB (umol/L: median, IQR)^1^** | 36.45 (25.325, 54.45) | 32.5 (21.7, 45.85) | 42.9 (30.8, 67.6) | 0.000 |
| **ALB (g/L: mean±SD)** | 32.918±3.6103 | 33.232±3.6993 | 32.611±3.5155 | 0.261 |
| **Scr (µmol/L: median, IQR)** | 70.5 (60, 87) | 68 (59, 81) | 74 (62, 101) | 0.021 |
| **CRP (mg/L: median, IQR)** | 13.65 (7.8, 26.925) | 12.6 (7.2, 19.6) | 15.4 (8.2, 29.2) | 0.206 |
| **PT (s: median, IQR)** | 14.9 (13.7, 16.675) | 14.4 (13.25, 15.25) | 15.6 (14.3, 17.2) | 0.000 |
| **D2 (g/L: median, IQR)** | 2.2 (1.8, 2.7) | 1.45 (0.923, 2.848) | 2.19 (1.335, 3.105) | 0.000 |
| **FIB (g/L: median, IQR)^2^** | 1.92 (1.015, 2.93) | 2.4 (2, 2.9) | 1.9 (1.5, 2.5) | 0.020 |
| **Peak PVV (cm/s: mean±SD)^3^** | 14.708±6.1358 | 16.409±6.2124 | 13.047±5.6134 | 0.000 |
| **HVPG (mmHg: mean±SD)** | 20.4129±5.34179 | 19.2323±3.96053 | 21.3748±6.15385 | 0.165 |
| **PVT (N, %)** | 12 (7.0%) | 5 (6.2%) | 7 (8.4%) | 0.578 |
| **Ascites grade (N, %)** |  |  |  | 0.533 |
| **Non** | 1 (0.6%) | 0 (0%) | 1 (1.1%) |  |
| **Grade 1 (mild)** | 4 (2.3%) | 2 (2.4%) | 2 (2.3%) |  |
| **Grade 2 (moderate)** | 123 (71.5%) | 58 (68.2%) | 65 (74.7%) |  |
| **Grade 3 (severe)** | 44 (25.6%) | 25 (29.4%) | 19 (21.8%) |  |

^1^TB: the normal range of TB in our center is < 20.5 umol/L.

^2^FIB: to note, two cases in the valid group had missing data (compared to three cases in the invalid group).

^3^Peak PVV: the normal range of peak PVV is ≥ 20 cm/s.
