# Supplementary material for: Development of a Drum Tower Severity Scoring (DTSS) system for pyrrolizidine alkaloid-induced hepatic sinusoidal obstruction syndrome
Source: Hepatol Int. 2022 Jan 12;16(3):669–79. doi: 10.1007/s12072-021-10293-5 (PMC9174127; doi:10.1007/s12072-021-10293-5)
Supplement: Supplementary file 1 — Supplementary file1 (ZIP 60 kb) [file 12072_2021_10293_MOESM1_ESM.zip › Table 2.docx]

Table 2 Logistic regression (the training set)

| **Variable** | **Univariate** | | | **Multivariate (Enter method)** | | |
| --- | --- | --- | --- | --- | --- | --- |
|  | **B** | **OR 95%CI** | **P** | **B** | **OR 95%CI** | **P** |
| **Acute onset** | -0.383 | 0.682[0.334,1.393] | 0.294 |  |  |  |
| **Time from onset to anticoagulation** | -0.009 | 0.991[0.997,1.005] | 0.209 |  |  |  |
| **ALT** | 0.003 | 1.003[1.000,1.005] | 0.056 |  |  |  |
| **AST** | 0.005 | 1.005[1.001,1.009] | 0.012 |  |  |  |
| **TB** | 0.029 | 1.030[1.012,1.048] | 0.001 | 0.031 | 1.032[1.004,1.061] | 0.026 |
| **ALB** | -0.047 | 0.955[0.869,1.049] | 0.333 |  |  |  |
| **Scr** | 0.008 | 1.008[0.997,1.021] | 0.166 |  |  |  |
| **PT** | 0.318 | 1.374[1.140,1.656] | 0.001 |  |  |  |
| **D2** | 0.106 | 1.112[0.933,1.324] | 0.235 |  |  |  |
| **FIB** | -0.899 | 0.407[0.224,0.741] | 0.003 |  |  |  |
| **Peak PVV** | -0.127 | 0.881[0.819,0.947] | 0.001 | -0.129 | 0.879[0.793,0.975] | 0.015 |
| **PVT** | 0.215 | 1.240[0.340,4.524] | 0.745 |  |  |  |
