# Supplementary material for: Development of a Drum Tower Severity Scoring (DTSS) system for pyrrolizidine alkaloid-induced hepatic sinusoidal obstruction syndrome
Source: Hepatol Int. 2022 Jan 12;16(3):669–79. doi: 10.1007/s12072-021-10293-5 (PMC9174127; doi:10.1007/s12072-021-10293-5)
Supplement: Supplementary file 1 — Supplementary file1 (ZIP 60 kb) [file 12072_2021_10293_MOESM1_ESM.zip › Table 3.docx]

Table 3 Performance of different models in subgroups

| **Model** | **The training set**  **(before anticoagulation)** | | | **The validation set**  **(before anticoagulation)** | | | **Revisited patients**  **(1 week anticoagulation)** | | | **Revisited patients**  **( 2 weeks anticoagulation)** | | |
| --- | --- | --- | --- | --- | --- | --- | --- | --- | --- | --- | --- | --- |
|  | **AUC**  **（95%CI）** | **SE^1^** | **P** | **AUC**  **（95%CI）** | **SE** | **P** | **AUC**  **（95%CI）** | **SE** | **P** | **AUC**  **（95%CI）** | **SE** | **P** |
| **①PT+FIB+AST**  **+TB+peak PVV+Scr** | 0.778  [0.696,0.859] | 0.042 | 0.000 | 0.775  [0.631,0.919] | 0.074 | 0.005 |  |  |  |  |  |  |
| **②AST+TB+peakPVV** | 0.768  [0.686,0.849] | 0.042 | 0.000 | 0.718  [0.561,0.875] | 0.080 | 0.022 |  |  |  |  |  |  |
| **③FIB+AST+TB**  **+peak PVV** | 0.773  [0.691,0.855] | 0.042 | 0.000 | 0.775  [0.628,0.922] | 0.075 | 0.005 | 0.840  [0.760,0.921] | 0.041 | 0.000 | 0.789  [0.672,0.906] | 0.060 | 0.000 |
| **④PT+FIB+AST**  **+TB+peak PVV** | 0.775  [0.693,0.856] | 0.042 | 0.000 | 0.775  [0.629,0.920] | 0.074 | 0.005 |  |  |  |  |  |  |
| **⑤DTSS system** | 0.787  [0.706,0.868] | 0.041 | 0.000 | 0.808  [0.670,0.946] | 0.070 | 0.002 | 0.812  [0.725,0.898] | 0.044 | 0.000 | 0.783  [0.663,0.902] | 0.061 | 0.000 |

^1^SE: standard error
