# Supplementary material for: Development of a Drum Tower Severity Scoring (DTSS) system for pyrrolizidine alkaloid-induced hepatic sinusoidal obstruction syndrome
Source: Hepatol Int. 2022 Jan 12;16(3):669–79. doi: 10.1007/s12072-021-10293-5 (PMC9174127; doi:10.1007/s12072-021-10293-5)
Supplement: Supplementary file 1 — Supplementary file1 (ZIP 60 kb) [file 12072_2021_10293_MOESM1_ESM.zip › Table 4.docx]

Table 4 Drum Tower Severity Scoring (DTSS) system

| **Variable** | **1 point** | **2 point** | **3 point** | **4 point** |
| --- | --- | --- | --- | --- |
| **AST(U/L)** | <69.75 | ≥69.75, <200 | ≥200, <320 | ≥320 |
| **TB (umol/L)** | <20.5 | ≥20.5, <38 | ≥38, <85.5 | ≥85.5 |
| **FIB (g/L)** | >2.35 | >1.5, ≤2.35 | >1, ≤1.5 | ≤1 |
| **Peak PVV (cm/s)** | ≥20 | >15.85, <20 | ≥10, ≤15.85 | <10 |
