# Supplementary material for: Development of a Drum Tower Severity Scoring (DTSS) system for pyrrolizidine alkaloid-induced hepatic sinusoidal obstruction syndrome
Source: Hepatol Int. 2022 Jan 12;16(3):669–79. doi: 10.1007/s12072-021-10293-5 (PMC9174127; doi:10.1007/s12072-021-10293-5)
Supplement: Supplementary file 1 — Supplementary file1 (ZIP 60 kb) [file 12072_2021_10293_MOESM1_ESM.zip › Table 5.docx]

Table 5 Diagnostic performance between DTSS system and revised EBMT criteria

|  | **Cut-off values** | **Ture**  **Positve**  **(a)** | **Fasle**  **Positive**  **(b)** | **Ture**  **Negative**  **(d)** | **Fasle**  **Negative**  **(c)** | **Sensitivity**  **(%)** | **Specificity**  **(%)** | **PPV**  **(%)** | **NPV**  **(%)** |
| --- | --- | --- | --- | --- | --- | --- | --- | --- | --- |
| **Training set**  **(DTSS, n = 127)** | 6.5 | 54 | 48 | 22 | 3 | 94.7% | 31.4% | 52.9% | 88% |
|  | 10.5 | 18 | 5 | 65 | 39 | 31.6% | 92.9% | 78.3% | 62.5% |
| **Validation set**  **(DTSS, n = 40)** | 6.5 | 25 | 10 | 3 | 2 | 92.6% | 23.1% | 71.4% | 60% |
|  | 10.5 | 8 | 0 | 13 | 19 | 29.6% | 100% | 100% | 40.6% |
| **One week Anticoagulation**  **(DTSS, n = 91)** | 6.5 | 36 | 24 | 27 | 4 | 90% | 52.9% | 60% | 87.1% |
|  | 10.5 | 8 | 1 | 50 | 32 | 20% | 98.0% | 88.9% | 61.0% |
| **Two weeks anticoagulation**  **(DTSS, n = 66)** | 6.5 | 23 | 15 | 24 | 4 | 85.2% | 61.5% | 60.5% | 85.7% |
|  | 10.5 | 4 | 0 | 39 | 23 | 14.8% | 100% | 100% | 62.9% |
| **Before anticoagulation**  **(EBMT*, n = 172)** | 1.5 | 42 | 37 | 48 | 45 | 48.3% | 56.5% | 53.2% | 51.6% |
|  | 2.5 | 20 | 4 | 81 | 67 | 23.0% | 95.3% | 83.3% | 54.7% |

*We redefined the modified EBMT classification: 1 for mild, 2 for moderate, 3 for severe, and 4 for very severe.
