# Supplementary material for: Development of a Drum Tower Severity Scoring (DTSS) system for pyrrolizidine alkaloid-induced hepatic sinusoidal obstruction syndrome
Source: Hepatol Int. 2022 Jan 12;16(3):669–79. doi: 10.1007/s12072-021-10293-5 (PMC9174127; doi:10.1007/s12072-021-10293-5)
Supplement: Supplementary file 3 — Supporting materials: including missing data and its disposal, a table of ROC analysis between related predictors, a table of RUCAM score to evaluate the drug-induced liver injury (DOCX 16 kb) [file 12072_2021_10293_MOESM3_ESM.docx]

**Part 1**

data missing in Table 1

Non-responder group: 22 patients underwent HVPG measurement, and the missing data included C-reactive protein in 2 cases, fibrinogen in 2 cases, D2 aggregates in 17 cases, and duration of medication in 1 case.

Responder group: 27 patients underwent HVPG measurement, and the missing data included fibrinogen in 3 cases, D2 aggregates in 10 cases, and the duration of medication was unknown in 2 cases.

To note, we thought that data missing in the DTSS system was only 5 cases of fibrinogen, and the quantification of indicators was not limited to the perspective of statistics. Therefore, for the sake of data authenticity, we did not make deletion and filling based statistics.

**Part 2**

ROC analysis (the training set)

| Variable | AUC (95%CI) | SE^1^ | P | Cut-off value | Sensitivity | Specificity | Youden index |
| --- | --- | --- | --- | --- | --- | --- | --- |
| Peak PVV (cm/s) | 0.680[0.589,0.772] | 0.047 | 0.000 | 15.85* | 75.9% | 56.3% | 0.322 |
| PT (s) | 0.672[0.578,0.767] | 0.048 | 0.001 | 15.05 | 60.3% | 71.8% | 0.321 |
| FIB (g/L) | 0.672[0.577,0.768] | 0.049 | 0.001 | 2.35* | 68.4% | 55.7% | 0.241 |
| TB (umol/L) | 0.657[0.561,0.753] | 0.049 | 0.002 | 38* | 56.9% | 67.6% | 0.245 |
| AST (U/L) | 0.645[0.548,0.742] | 0.049 | 0.005 | 69.75* | 74.1% | 53.5% | 0.276 |
| Scr (mmol/L) | 0.620[0.522,0.718] | 0.050 | 0.019 | 89.5 | 32.8% | 88.7% | 0.215 |

^1^SE: standard error

*cut-off values marked by red color were used to design DTSS system.

**Part 3**

RUCAM score

|  | Valid (n=85) | Invalid (n=87) |
| --- | --- | --- |
| 3-5 points (possible) | 17 (20%) | 11 (12.64%) |
| 6-8 points (great possible) | 64 (75.29%) | 67 (77.01%) |
| > 8points (highly possible) | 4 (4.71%) | 9 (10.34%) |
